# Multiresistant extended-spectrum β-lactamase-producing *Enterobacteriaceae* from humans, companion animals and horses in central Hesse, Germany

**DOI:** 10.1186/1471-2180-14-187

**Published:** 2014-07-12

**Authors:** Judith Schmiedel, Linda Falgenhauer, Eugen Domann, Rolf Bauerfeind, Ellen Prenger-Berninghoff, Can Imirzalioglu, Trinad Chakraborty

**Affiliations:** 1 Institute of Medical Microbiology, Justus Liebig University Giessen and German Center for Infection Research (DZIF), Partner site Giessen-Marburg-Langen, Schubertstrasse 81, 35392 Giessen, Germany; 2 Institute of Hygiene and Infectious Diseases of Animals, Justus Liebig University Giessen, Frankfurter Strasse 85-89, 35392 Giessen, Germany

**Keywords:** ESBL, *Enterobacteriaceae*, Comparison, Human isolates, Animal isolates, One Health concept

## Abstract

**Background:**

Multiresistant Gram-negative bacteria producing extended-spectrum β-lactamases (ESBLs) are an emerging problem in human and veterinary medicine. This study focused on comparative molecular characterization of β-lactamase and ESBL-producing *Enterobacteriaceae* isolates from central Hesse in Germany. Isolates originated from humans, companion animals (dogs and cats) and horses.

**Results:**

In this study 153 (83.6%) of the human isolates (n = 183) and 163 (91.6%) of the animal isolates (n = 178) were confirmed as ESBL producers by PCR and subsequent sequencing of the PCR amplicons. Predominant ESBL subtypes in human and animal samples were CTX-M-15 (49.3%) and CTX-M-1 (25.8%) respectively. Subtype *bla*_CTX-M-2_ was found almost exclusively in equine and was absent from human isolates. The carbapenemase OXA-48 was detected in 19 ertapenem-resistant companion animal isolates in this study. The Plasmid-encoded quinolone resistance (PMQR) gene *aac(‘6)-Ib-cr* was the most frequently detected antibiotic- resistance gene present in 27.9% of the human and 36.9% of the animal ciprofloxacin-resistant isolates. Combinations of two or up to six different resistance genes (penicillinases, ESBLs and PMQR) were detected in 70% of all isolates investigated. The most frequent species in this study was *Escherichia coli* (74%), followed by *Klebsiella pneumoniae* (17.5%), and *Enterobacter cloacae* (4.2%). Investigation of *Escherichia coli* phylogenetic groups revealed underrepresentation of group B2 within the animal isolates.

**Conclusions:**

Isolates from human, companion animals and horses shared several characteristics regarding presence of ESBL, PMQR and combination of different resistance genes. The results indicate active transmission and dissemination of multi-resistant *Enterobacteriaceae* among human and animal populations.

## Background

Extended-spectrum beta-lactamase (ESBL)-producing *Enterobacteriaceae* are isolated with increasing frequency from human and animal samples. In particular, the species *Escherichia (E.) coli* plays an important role as a source of the corresponding resistance genes [1]. ESBLs genes are commonly plasmid-encoded and can easily be transmitted by conjugation to other bacteria, even across species barriers. In addition, various resistance genes are located in or close to mobile genetic elements such as insertion sequences and transposons [2]. Lateral gene transfer and continuous DNA recombination make it extremely difficult to track transmission pathways of ESBL genes in bacterial populations. The transmission of ESBL-producing pathogens or ESBL genes between companion animals/livestock and owner/caretaker/consumer is currently a subject of intense and controversial discussion. Evidence has been presented for zoonotic spread [3,4]. Several studies have addressed the risk of infection that could arise from keeping animals [5-7]. A recent study highlighted that the risk factors for ESBL carriage were travel to Greece or Africa within the last 12 months and keeping of pet animals, but not antibiotic consumption or recent hospitalization [8]. Until now, it is still difficult to estimate the amount of exchange and even more difficult to define the risk for human and animal health and also food safety.

The focus of this study was a comparative investigation of 361 ESBL-producing *Enterobacteriaceae* isolates obtained from animals and human patients presenting at a veterinary clinic and a hospital, respectively, both serving a similar catchment area. The prevalence of β-lactamase-, particularly ESBL-producing bacteria in companion animal, horse and human isolates of clinical origin was examined. Resistance genes (β-lactamase and plasmid-mediated quinolone resistance (PMQR)) and *E. coli* phylogenetic groups were investigated using molecular methods.

This work sheds light on shared populations of ESBL-producing *Enterobacteriaceae* in symptomatic companion animals, horses and humans in the geographical region of middle Hesse, Germany.

## Methods

### Bacterial isolates

A collection of 513 *Enterobacteriaceae* isolates resistant to one or more third-generation cephalosporins (200 human isolates and 313 animal isolates) was examined. Identification was done using in-house biochemical tests. In case of ambiguous results confirmation was done using API 20E. Prior to further analysis, all isolates were grown on MacConkey agar supplemented with cefotaxime (2 mg/l) to promote selection of β-lactamase producers and ensure selection of *Enterobacteriaceae*. Afterwards isolates were tested for possible ESBL production by double disc synergy test (DDST) according to EUCAST guidelines for resistance mechanisms [9]. After performance of the DDST 183 human and 178 animal isolates were categorized as possible ESBL-producers and forwarded for a more detailed investigation. Isolates with a negative DDST result were not included in the study.

DDST-positive isolates from human clinical samples (n = 183) were taken from the strain collection of the Institute of Medical Microbiology, Giessen. Some of the isolates originate from routine screening for colonization with ESBL-producing *Enterobacteriaceae* at the University hospital Giessen. Additional isolates were collected from samples of clinically ill patients e.g. blood culture or urine samples in the same facility. All isolates were collected between 2009 and 2010. Approximately two-thirds of the human isolates originated from inpatients (n = 128) and one third were isolates from outpatients wards (n = 55).

DDST-positive isolates from animals (n = 178) were obtained during a survey for aerobic Gram-negative bacteria growing on MacConkey agar supplemented with cefotaxime (2 mg/l) among animal patients at the veterinary clinics of the Justus Liebig University (JLU) Giessen. All samples were clinically relevant infection related isolates. The isolates originated from horses (n = 100), dogs (n = 67) and cats (n = 11) and were collected between 2009 and 2011.

The study was approved by the ethics committee of medical faculty of the Justus Liebig University of Giessen and deemed exempt from informed consent.

### Antibiotic susceptibility

Antibiotic susceptibility testing was done using the VITEK®2 compact system with AST N117 cards (Biomérieux) and Etest® stripes (Liofilchem®) containing ertapenem, cefepime, chloramphenicol and nalidixic acid. Results were evaluated according to CLSI guidelines for human pathogens (CLSI, 2012).

### β-lactamase identification

For antibiotic resistance genes screening by PCR and identification by sequencing were performed as described previously [10]. Specific oligonucleotide primers for *bla*_TEM_, *bla*_SHV_[11], *bla*_CTX-M_ genes [12] and *bla*_OXA-1_[13] were used. The MAST carbapenemase detection set (MAST group, UK) was applied on carbapenem-resistant isolates. Furthermore, primers for *bla*_OXA-48_ were used for carbapenem-resistant isolates [11]. With the exception of *bla*_OXA-1_-positive strains, amplicons of PCR-positive strains were sequenced to identify the encoded ESBL allele in detail. Sequencing was performed using the automated sequencer ABI Prism® 3100 (Life technologies, USA). The blastn algorithm of NCBI (http://www.ncbi.nlm.nih.gov/blast/) was used for database searches to identify the resistance gene allele. The primers and the sequences they were derived from are presented in Table 1. Positive control strains from the Institute of Microbiology, Giessen, Germany, were *Klebsiella* (*K.*) *pneumoniae* H59 for *bla*_TEM_, *bla*_SHV_, *bla*_CTX-M_, *bla*_OXA-1_ and *aac(6’)-Ib*, *Klebsiella pneumoniae* 714 for *bla*_OXA-48_. For the *qnr*-genes, strains from the Institute of Hygiene and Infectious Diseases of Animals, Giessen, Germany (V148 for *qnrA*, V167 for *qnrB*, V60 for *qnrD* and V61 for *qnrS)*, were used as positive controls after sequencing and comparison with known sequences using DNASTAR software (DNASTAR Inc, Madison, USA) and the NCBI blastn algorithm.

**Table 1:** **PCR primers used to detect β-lactamase genes, PMQR genes and *E. coli *** **phylogenetic groups**

**Primer**	**Sequence (5’ - 3’)**	**Target**	**Size of product (bp)**	**Reference**
TEM_f	ATGAGTATTCAACATTTCCG	*bla*_TEM_	851	[11]
TEM_r	TTAATCAGTGAGGCACCTAT			
SHV_f	GCAAAACGCCGGGTTATTC	*bla*_SHV_	940	[11]
SHV_r	GGTTAGCGTTGCCAGTGCT			
CTX-M_f	TCTTCCAGAATAAGGAATCCC	*bla*_CTX-M_	909	[12]
CTX-M_r	CCGTTTCCGCTATTACAAAC			
OXA-1_f	AGCAGCGCCAGTGCATCA	*bla*_OXA-1_	700	[13]
OXA-1_r	ATTCGACCCCAAGTTTCC			
OXA-48_f	AAATCACAGGGCGTAGTTGTG	*bla*_OXA-48_	555	[11]
OXA-48_r	GACCCACCAGCCAATCTTAG			
qnrA_f	AGAGGATTTCTCACGCCAGG	*qnrA*	619	[14]
qnrA_r	GCAGCACTATKACTCCCAAGG			
qnrB_f	GGMATHGAAATTCGCCACTG	*qnrB*	264	[16]
qnrB_r	TTTGCYGYYCGCCAGTCGAA			
qnrC_f	GGGTTGTACATTTATTGAATC	*qnrC*	447	[17]
qnrC_r	TCCACTTTACGAGGTTCT			
qnrD_f	CGAGATCAATTTACGGGGAATA	*qnrD*	582	[18]
qnrD_r	AACAAGCTGAAGCGCCTG			
qnrS_f	GCAAGTTCATTGAACAGGCT	*qnrS*	428	[16]
qnrS_r	TCTAAACCGTCGAGTTCGGCG			
qepA_f	CTGCAGGTACTGCGTCATG	*qepA*	403	[15]
qepA_r	CGTGTTGCTGGAGTTCTTC			
aac_f	TTGCGATGCTCTATGAGTGGCTA	*aac(6’)-Ib*	482	[19]
aac_r	CTCGAATGCCTGGCGTGTTT			
chuA.1	GACGAACCAACGGTCAGGAT	*chuA*	279	[21]
chuA.2	TGCCGCCAGTACCAAAGACA			
YjaA.1	TGAAGTGTCAGGAGACGCTG	*yajA*	211	[21]
YjaA.2	ATGGAGAATGCGTTCCTCAAC			
TspE4C2.1	GAGTAATGTCGGGGCATTCA	TspE4C2	152	[21]
TspE4C2.2	CGCGCCAACAAAGTATTACG			

### Detection of Plasmid-encoded quinolone resistance (PMQR)

Detection for PMQR by PCR was performed on all isolates with a minimum inhibitory concentration (MIC) ≥ 1 μg/ml for ciprofloxacin (Human isolates: n = 140, animal isolates: n = 122). Oligonucleotide primers for detection of *qnrA, qnrB, qnrC, qnrD, qnrS, qepA and aac(6’)-Ib* were used [14-19]. Identification of the *aac(6’)-Ib-cr* variant was performed according to Jones et al. 2008 [20].

### Phylogenetic groups of ESBL-producers

Distribution of *E. coli* phylogenetic groups was analysed by PCR targeting genes *chuA*, *yjaA* and the DNA fragment TSPE4.C2 to assign the strains to the groups A, B1, B2 and D according to Clermont et al. 2000 [21].

### Analysis of resistance genes patterns

Information regarding the source of isolates, species and the resistance genes detected were assembled into an Excel file. The data was subsequently analysed using GENE-E [22] which enables the clustering of data with matrix visualization and analysis to support visual data exploration.

## Results

### Overview of bacterial species

The majority of the isolates studied were *E. coli* (74%), followed by *K. pneumoniae* (17.5%) and *Enterobacter cloacae* (4.2%). Other species detected were *Klebsiella oxytoca*, *Enterobacter intermedius*, *Citrobacter freundii*, *Providencia stuartii, Morganella morganii* and *Proteus mirabilis*. The percentages of *E. coli* and *K. pneumoniae* isolates among the human and animal isolates were very similar.

### Resistance rates

All isolates (n = 361) were categorised as multidrug resistant (MDR) according to the international expert proposal for interim standard definitions for acquired resistance promoted by the ECDC [23]. Both human (n = 183) and animal isolates (n = 178) revealed resistance against ampicillin and trimethoprim/sulfmethoxazole. Slight differences could be observed concerning the tested third generation cephalosporins ceftazidime, cefotaxime and cefepime. Of the human isolates 52.5% (n = 96) showed resistance against ceftazidime, 78.7% (n = 144) resistance against cefotaxime and 98.4% (n = 180) resistance against cefepime. Of the animal isolates 75.8% (n = 135) displayed resistance against ceftazidime and 99.4% (n = 177) to cefotaxime and cefepime. Of the human isolates 2.2% (n = 4), and 1.1% (n = 2) of the animal isolates revealed resistance against imipenem. Resistance against the other tested carbapenem (ertapenem) was detected in 24.5% (n = 45) of human isolates and in 19.7% (n = 35) of animal isolates. The aminoglycosides gentamicin and amikacin differed in their results. Only 5.1% (n = 9) of animal isolates exhibited resistance against amikacin, as compared to 14.8% (n = 27) of the human isolates. The resistance rates against gentamicin were higher in both cases with 35% (n = 64) in human isolates and 62.4% (n = 111) in animal isolates. High resistance rates in both groups were also revealed for the fluoroquinolone ciprofloxacin (76.5% (n = 140) of the human isolates and 68.5% (n = 122) of the animal isolates).

### Distribution of β-lactamases

Almost all isolates carried at least one beta-lactamase (*bla)*-gene (Table 2). In all, 91.6% of the animal isolates and 83.6% of the human isolates were confirmed as ESBL producers by PCR and sequencing. The remaining isolates (7.5%) were negative for the presence of *bla*_TEM_, *bla*_SHV_, *bla*_OXA-1_, *bla*_OXA-48_ and *bla*_CTX-M_.

**Table 2:** **Distribution of β-lactamase genes in *Enterobacteriaceae *** **isolates (n = 361) of human and animal origin**

**Source**	**Human**		**Animal**			
	**Outpatients**	**Inpatients**	**Dogs**	**Cats**	**Horses**	**Total no. of isolates**
**No. of isolates**^**a**^	55	128	67	11	100	361
**Penicillinases**^**b**^						
*bla*_TEM-1_	58.2 (32)	41.4 (53)	62.7 (42)	45.5 (5)	67 (67)	55.1 (199)
*bla*_TEM-190_			1.5 (1)			0.3 (1)
*bla*_SHV-1_		2.3 (3)	16.4 (11)		2 (2)	4.4 (16)
*bla*_SHV-11_		1.6 (2)				0.6 (2)
*bla*_OXA-1_	23.6 (13)	32.8 (42)	59.7 (40)	45.5 (5)	29 (29)	35.7 (129)
**ESBL**^**b**^						
*bla*_TEM-52_		2.3 (3)				0.8 (3)
*bla*_SHV-2_			1.5 (1)	9.1 (1)		0.6 (2)
*bla*_SHV-5_		0.8 (1)				0.3 (1)
*bla*_SHV-28_			3(2)		1(1)	0.8 (3)
*bla*_CTX-M-1_	32.7 (18)	45.3 (24)	16.4 (11)	27.3 (3)	37 (37)	25.8 (93)
*bla*_CTX-M-2_				9.1 (1)	15 (15)	4.4 (16)
*bla*_CTX-M-9_			3 (2)	9.1 (1)	1 (1)	1.1 (4)
*bla*_CTX-M-14_			1.5 (1)			0.3 (1)
*bla*_CTX-M-15_	54.5 (30)	51.6 (66)	59.7 (40)	36.4 (4)	38 (38)	49.3 (178)
*bla*_CTX-M-32_	1.8 (1)	0.8 (1)				0.6 (2)
*bla*_CTX-M-38_		0.8 (1)				0.3 (1)
*bla*_CTX-M-79_	1.8 (1)	2.3 (3)			1(1)	1.4 (5)
*bla*_CTX-M-97_	1.8 (1)				3(3)	1.1 (4)
*bla*_CTX-M-117_		0.8 (1)				0.3 (1)
**Carbapenemases**^**b**^						
*bla*_OXA-48_			23.9 (16)	18.2 (2)	1(1)	5.3 (19)

^a^numerous isolates encoded for more than one β-lactamase gene.

^b^% of β-lactamase genes (no. of isolates).

### CTX-M

**Figure 1:**
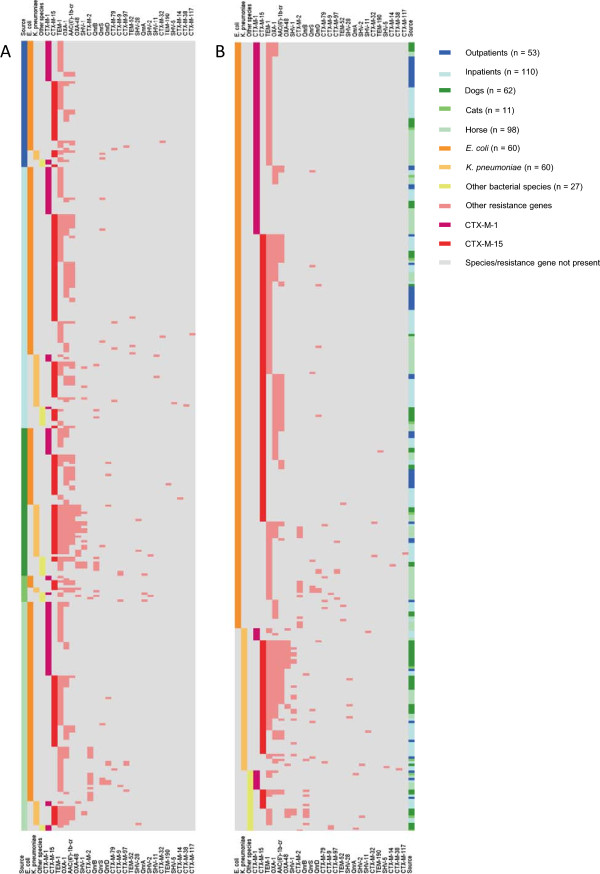
**Heat maps generated from identified resistance genes and bacterial species among human and animal isolates.** Identified resistance genes and bacterial species are listed according to frequency (except for CTX-M-1 and CTX-M-15) on the right and left side of the figures. Source = origin of isolates (outpatients, inpatients, dogs, cats, horses). Not included are isolates without detectable resistance gene (n = 28). The term “Other species” includes *Enterobacter cloacae* (n = 12), *Klebsiella oxytoca* (n = 9), *Enterobacter intermedius* (n = 2), *Enterobacter gergoviae* (n = 1), *Citrobacter freundii* (n = 1) and *Proteus mirabilis* (n = 1). **A** is focussing on the origin of isolates whereas the **B** emphasizes the involved bacterial species.

Subtypes of the ESBL CTX-M were by far the most frequently encoded ESBLs in both groups (Table 2 and Figure 1). Among the human isolates 23% encoded CTX-M-1 and 52.5% CTX-M-15. Some human isolates carried *bla*_CTX-M-79_, *bla*_CTX-M-32_, *bla*_CTX-M-97_, *bla*_CTX-M-38_ or *bla*_CTX-M-117_. The *bla*_CTX-M-1_ gene was found in 32.7% of human outpatient isolates and in 45.3% of inpatient isolates, while prevalence rates of *bla*_CTX-M-15_ were high and similar in both groups (outpatients 54.5%, inpatients 51.6%). Animal isolates revealed similar results as 28.7% of these isolates carried *bla*_CTX-M-1_ and 46.1% *bla*_CTX-M-15_. Other detected *bla*_CTX-M_ genes were *bla*_CTX-M-2_, *bla*_CTX-M-9_, *bla*_CTX-M-97_, *bla*_CTX-M-79_, and *bla*_CTX-M-14_. The presence of *bla*_CTX-M-2,_* bla*_CTX-M-9_, and *bla*_CTX-M-14_ was restricted to animal isolates, while *bla*_CTX-M-32_, *bla*_CTX-M-38_, *bla*_CTX-M-117_ were found only among the human isolates. *Bla*_CTX-M-1_ was the dominant resistance subtype in horses (37%) but it was less frequently isolated in dogs (16.4%). In contrast, *bla*_CTX-M-15_ occurred more often in dog isolates (59.7%) than in isolates from horses (38%) or cats (36.4%). In addition, *bla*_CTX-M-15_ was also the dominant CTX-M subtype among *K. pneumoniae* isolates whereas among the *E. coli* isolates both subtypes were present to the same degree. Altogether, *bla*_CTX-M-1_ and *bla*_CTX-M-15_ were the prominent β-lactamase genes present in 25.8% and 49.3% of all investigated isolates.

### TEM

Non-ESBL gene *bla*_TEM-1_ was the most frequently identified β-lactamase gene among all investigated isolates. It was detected in 46.4% of the human isolates and in 64% of all animal isolates. It was present in 199 of 361 isolates (55%). The highest detection rates were observed in outpatients (58.2%), inpatients (41.4%), dog isolates (62.7%) and horse isolates (67%). TEM-52 was the only TEM-type ESBL that occurred in this study. It was identified in three isolates from human inpatients. Results are shown in Table 2.

### SHV

Prevalence of *bla*_SHV_ was 17.2% in human and 52.9% in animal *K. pneumoniae. Bla*_SHV-1_ was identified in 10.3% of the human and in 29.4% of the animal *K. pneumoniae* isolates. Several ESBL variants could be identified in addition (Table 2). Most of the latter isolates originated from dogs. Detected SHV-type ESBL genes were *bla*_SHV-2_, *bla*_SHV-5_, *bla*_SHV-11_ and *bla*_SHV-28_. *Bla*_SHV-5_ and *bla*_SHV-11_ were restricted to isolates from human inpatients. ESBL genes *bla*_SHV-2_ and *bla*_SHV-28_ were detected among the animal isolates only.

### OXA-1

The presence of the penicillinase gene *bla*_OXA-1_ could be demonstrated in 30.1% of the human isolates. Among the animal isolates *bla*_OXA-1_ was identified in 41.6% of all strains. Detection rates were highest in isolates from dogs (59.7%), followed by cat (45.5%), outpatient (32.8%) and horse (29%) isolates. However, *bla*_OXA-1_ was also found in inpatient isolates (23.6%) (Table 2).

### OXA-48

All carbapenem resistant isolates (human isolates: 45, animal isolates: 40) were tested for presence of the carbapenemase OXA-48. None of the tested human isolates harboured *bla*_OXA-48_. In contrast, 19 (5.3%) of the animal isolates harboured this gene. The isolates harbouring *bla*_OXA-48_ originated from dogs (16), cats (2) and a horse (Table 2).

### PMQR

Distribution and presence of PMQR genes are illustrated in Table 3. The most frequently detected PMQR gene in ciprofloxacin-resistant isolates (n = 262) was *aac(‘6)-Ib-cr*. Prevalence was 27.9% (n = 39) in the human isolates, 36.9% (n = 45) in the animal isolates and 32.1% (n = 84) in all investigated isolates. None of the isolates carried *qnrC* or *qepA*. Among the human isolates *qnrB* was found in three and *qnrS* in five isolates. Two more *qnr* variants were detected among the animal isolates. Gene *qnrA* was carried by three, *qnrB* by seven, *qnrD* by eight and *qnrS* by five animal isolates. Most *qnr* type genes (*qnrA, qnrB, qnrD, qnrS*) were detected in dog isolates. Inpatient isolates carried PMQR genes at higher rates (37.8%) than isolates from outpatients (23.8%). A similar observation could be made among the animal isolates. Highest identification rates (49.5%) were observed in isolates originating from animals tested during stay in the small animal clinic. In summary, most PMQR genes were present in dog isolates (65.1%). Overall, PMQR genes could be demonstrated in 43.9% (n = 115) of all investigated isolates.

**Table 3:** Distribution of PMQR genes in ciprofloxacin-resistant isolates (n = 262) of human and animal origin

		**% of PMQR genes (no. of isolates)**						
**Source**	**No. of isolates**^**b**^	***qnrA***	***qnrB***	***qnrC***	***qnrD***	***qnrS***	***qepA***	***aac(6’)-Ib-cr***
**Human**								
Outpatients	42					4.8 (2)		19 (8)
Inpatients	98		3.1 (3)			3.1 (3)		31.6 (31)
Total	140		2.1 (3)			3.6 (5)		27.9 (39)
**Animal**								
Dogs	63	1.6 (1)	7.9 (5)		4.8 (3)	1.6 (1)		49.2 (31)
Cats	10	20 (2)	20 (2)					40 (4)
Horses	49				10.2 (5)	8.2 (4)		20.4 (10)
Total	122	2.5 (3)	5.7 (7)		6.6 (8)	4.1 (5)		36.9 (45)
**Total no. of isolates**	262	1.1 (3)	3.8 (10)		3.1 (8)	3.8 (10)		32.1 (84)

^b^some isolates encoded for more than one PMQR gene.

### Analysis of resistance gene patterns

Preliminary detection and visualization of clusters and/or resistance patterns in context to isolate source and bacterial species was performed using Figure 1 which was generated by means of the GENE-E program. Data was then confirmed by analysis of absolute and relative numbers/ percentages as displayed in Table 4. Combinations of various plasmid encoded resistance genes (ESBL and non-ESBL β-lactamases and PMQR) were observed in the majority of isolates (70.1%) included in this study (Table 4 and Figure 1). Most frequently detected (26.3%) was the combination of a TEM type penicillinase with a CTX-M type ESBL. Also regularly observed was a penicillinase (TEM or OXA-1) in combination with CTX-M and plasmid-mediated quinolone resistance (PMQR) genes. These combinations were predominantly found among *E. coli* isolates. Other isolates encoded for several combinations of a TEM penicillinase, CTX-M enzymes, the penicillinase *bla*_OXA-1_, the penicillinase *bla*_SHV_ and PMQR genes. The carbapenemase OXA-48 was mainly associated with numerous resistance genes such as a TEM penicillinase, CTX-M and PMQR. In addition, it was exclusively identified in *K. pneumoniae* and *Enterobacter cloacae* isolates from animals. Another frequently observed combination was that of *bla*_CTX-M-15_ and *aac(‘6)-Ib-cr*. Other combinations were detected only once or to a much lesser extent than those mentioned above. Combination of more than two resistance genes could be observed more often among human *E. coli* isolates and especially among *K. pneumoniae* animal isolates (Figure 1).

**Table 4:** Observed combinations of β-lactamase and PMQR genes according to bacterial species

**Combination of resistance genes**^**a**^	**Outpatients**					**Inpatients**					**Dogs**					**Cats**					**Horses**				
	**% of bacterial species (no. of isolates)**																								
	***EC***	***KP***	***KO***	***EN***	***OS***^***b***^	***EC***	***KP***	***KO***	***EN***	***OS***^***b***^	***EC***	***KP***	***KO***	***EN***	***OS***^***b***^	***EC***	***KP***	***KO***	***EN***	***OS***^***b***^	***EC***	***KP***	***KO***	***EN***	***OS***^***b***^
**TEM**_**Pen**_**/CTX-M**	50 (24)					26.9 (25)	12 (3)				17.1 (6)					20 (1)					40.7 (35)				25 (1)
**TEM**_**Pen**_**/CTX-M/PMQR**		25 (1)				1.2 (1)			25 (1)		2.9 (1)					20 (1)			33.3 (1)		8.1 (7)				
**TEM**_**Pen**_**/CTX-M/OXA**_**Pen**_	2.1 (1)		33.3 (1)			2.2 (2)					14.3 (5)										12.8 (11)	10 (1)			
**TEM**_**Pen**_**/CTX-M/OXA**_**Pen**_**/PMQR**	4.2 (2)	25 (1)				8.6 (8)	8 (2)				8.6 (3)	18.2 (4)	100 (1)	12.5 (1)		20 (1)					1.2 (1)	40 (4)			
**TEM**_**Pen**_**/SHV**_**Pen**_**/CTX-M/OXA**_**Pen**_**/PMQR**							4 (1)					9.1 (2)										20 (2)			
**TEM**_**Pen**_**/SHV**_**Pen**_**/CTX-M/OXA**_**Pen**_**/OXA**_**Carba**_**/PMQR**												22.7 (5)													
**TEM**_**Pen**_**/CTX-M/OXA**_**Pen**_**/OXA**_**Carba**_**/PMQR**												27.3 (6)					50 (1)								
**CTX-M/OXA**_**Pen**_**/PMQR**	8.3 (4)					14 (13)	12 (3)				17.1 (6)					20 (1)					3.5 (3)				
**TEM**_**Pen**_**/SHV**_**Pen**_**/OXA**_**Carba**_**/PMQR**														37.5 (3)											
**CTX-M/PMQR**						2.2 (2)		20 (1)			5.7 (2)			12.5 (1)					33.3 (1)						
**CTX-M/OXA**_**Pen**_	8.3 (4)					6.5 (6)	8 (2)				2.9 (1)					20 (1)					8.1 (7)				
**Other combinations**	2.1 (1)	25 (1)				3.2 (3)	16 (4)					22.7 (5)		12.5 (1)			50 (1)		33.3 (1)			20 (2)			
**No resistance gene detected**	4.2 (2)					14 (13)	12 (3)		50 (2)		8.6 (3)			12.5 (1)	100 (1)						2.3 (2)				
**Single resistance gene**	20.8 (10)	25 (1)	66.7 (2)			21.5 (20)	28 (7)	80 (4)	25 (1)	100 (1)	22.9 (8)			12.5 (1)		20 (1)				1	23.3 (20)	10 (1)			75 (3)
**Total no. of isolates**	48	4	3	0	0	93	25	5	4	1	35	22	1	8	1	5	2	0	3	1	86	10	0	0	4

EC, Escherichia coli; KP, Klebsiella pneumoniae; KO, Klebsiella oxytoca; EN, Enterobacter cloacae; OS, Other species.

^a^TEM_Pen_, TEM Penicillinase, OXA_Pen_, OXA Pencillinase, OXA_Carba_, OXA Carbapenemase, SHV_Pen_, SHV Penicillinase.

^b^Other species include *Enterobacter intermedius* (n = 2). *Enterobacter gergoviae* (n = 1), *Citrobacter freundii* (n = 1), and *Proteus mirabilis* (n = 1), *Providencia stuartii* (n = 1), *Morganella morganii* (n = 1).

### Phylogenetic grouping of β-lactamase-producing *E. coli*

**Figure 2:**
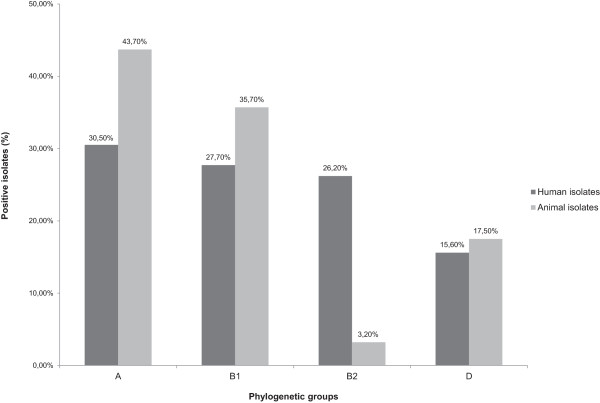
**Distribution of *E. coli *phylogenetic groups among human and animal ESBL-producing isolates.** A total of 267 isolates from human (n = 141) and animal (n = 126) sources were assigned to *E. coli* phylogenetic groups A, B1, B2 and D.

Phylogenetic group typing was applied to 141 human and 126 animal ESBL-producing *E. coli* isolates (Table 5 and Figure 2). Both groups differed from each other in the frequencies of known phylogenetic groups. In general, the human isolates were almost evenly distributed among the phylogenetic groups A (30.5%), B1 (27.7%) and B2 (26.2%) and to a lesser extent with group D (15.6%). In contrast, most of the analysed animal isolates belonged to group A (43.7%) and a considerably smaller number of isolates to B1 (35.7%). Approximately 17.5% of all animal isolates could be assigned to group D, whereas only 2.4% were classified as group B2.

**Table 5:** **Distribution of *E. coli *** **phylogenetic groups among human and animal isolates (n = 267)**

		**% of phylogenetic groups (no. of isolates)**			
**Source**	**No. of isolates**	**A**	**B1**	**B2**	**D**
**Human**					
Outpatients	48	31.3 (15)	29.2 (14)	25 (12)	14.6 (7)
Inpatients	93	30.1 (28)	26.9 (25)	26.9 (25)	16.1 (15)
Total	141	30.5 (43)	27.7 (39)	26.2 (37)	15.6 (22)
**Animal**					
Dogs	35	48.6 (17)	22.9 (8)	2.9 (1)	25.7 (9)
Cats	5	60 (3)	20 (1)		20 (1)
Horses	86	40.7 (35)	41.9 (36)	2.3 (2)	14 (12)
Total	126	43.7 (55)	35.7 (45)	2.4 (3)	17.5 (22)
**Total no. of isolates**	267	36.7 (98)	31.5 (84)	14.9 (40)	16.5 (44)

## Discussion

The global spread of multi-resistant ESBL-producing *Enterobacteriaceae* can in part be explained by mobility of the resistance bearing genetic elements e.g. plasmids, transposons and insertion sequence elements. This fact is a major concern in terms of epidemiology and infection control. Transmission pathways between humans and animals are currently a subject of active discussion [8,24,25]. This study was designed to investigate the distribution of β-lactamases, particularly ESBL, and PMQR genes among isolates from human, companion animal and horse samples in a defined geographical area of Germany. The key question was whether similar resistance characteristics are currently present among both groups. Indeed, our studies here demonstrate that isolates from human and animal samples share numerous characteristics.

Antimicrobial susceptibility testing revealed common and disturbing patterns. High resistance rates for all β-lactams and other antibiotic classes were observed. In particular animal isolates harbouring resistance to the last-resort antibiotics ertapenem and impenem were detected. The results for ertapenem were particularly alarming, with 16.3% resistance among animal isolates and 24% among the human isolates. In Germany, the use of carbapenems in livestock animals is prohibited and its use for treating companion animals is strongly restricted. Nevertheless, almost all ertapenem resistant strains among the animal isolates (95.6%) originated from dogs and cats. This would suggest human to animal transmission of resistant strains by their owners. Alternatively, the use of carbapenems for treatment of companion animals cannot be excluded even though these pharmaceuticals are expensive and their use is uncommon even in pet animal practice. Carbapenemase activity in bacteria from companion animals and livestock has been already identified recently [26-28]. To investigate this phenomenon in more detail, all carbapenem resistant isolates from this study were tested for the presence of *bla*_OXA-48_. Surprisingly, a substantial difference could be observed regarding the molecular mechanisms underlying this resistance phenotype. None of the 45 tested human isolates was positive for *bla*_OXA-48_, though 26 (57.8%) were identified with a possible combination of an AmpC enzyme and porin deficiency. This mechanism has been observed previously in AmpC-producing *K. pneumoniae* by Shi et al. They stated that the combination of AmpC production and porin loss can result in reduced susceptibility to carbapenems [29]. In contrast to these results, 19 (47.5%) of the 40 examined animal isolates harboured an OXA-48 carbapenemase.

Among 361 isolates resistant to one or more third generation cephalosporins 84% harboured a CTX-M type enzyme. Predominant ESBL genes in human and animal samples were *bla*_CTX-M-15_ and *bla*_CTX-M-1_. In the past *bla*_CTX-M-15_ was merely associated with isolates from humans [30] and CTX-M-1 was the major CTX-M subtype in cattle and pigs in Europe [31]. In addition *bla*_CTX-M-1_ was also detected in companion animal and poultry isolates [32,33]. In the meantime, attribution of the above-named CTX-M types as specific to either human or animal isolates is no longer tenable. An increasing number of studies have identified *bla*_CTX-M-1_ and *bla*_CTX-M-15_ in both populations [3,34] and our study confirmed this development. Among our isolates *bla*_CTX-M-15_ was the most prevalent ESBL gene which occurred in approximately 50% of the strains investigated (human: 52.5%, animal: 46.1%). The number for *bla*_CTX-M-1_ was considerably lesser but this gene was still relatively frequently present (23%, 28.7%). Isolates collected from horses seemed to hold a special position since rates of *bla*_CTX-M-1_- and *bla*_CTX-M-15_-positive isolates were almost equal (*bla*_CTX-M-1_ 37%; *bla*_CTX-M-15_ 38%). Previous studies have mainly identified CTX-M-1 as the predominant ESBL subtype among horse isolates [35,36], which is also the most common type among livestock. In Germany horses are mostly used as recreational animals, normally situated close to livestock or in livestock-like environments. However, they usually have closer contact to humans and in case of disease medical care for horses is much more intense than in agricultural animal species. This could be a possible explanation for the particularly high detection rates of *bla*_CTX-M-15_ reported here. Interestingly, *bla*_CTX-M-2_ was found almost exclusively in equine *E. coli* isolates and was not present among human isolates. A study from Belgium identified *bla*_CTX-M-2_ as major CTX-M subtype among diseased horses [37]. These animals had received prolonged treatment with several antibiotics and the *bla*_CTX-M-2_ gene was part of the novel complex class 1 integron IS*CR*_1_. Ferreira et al. could demonstrate the same linkage between *bla*_CTX-M-2_ and IS*CR*_1_ however, within the chromosome of *E. coli* isolates from healthy broiler chickens in Brazil [38]. Further investigation of the region surrounding the *bla*_CTX-M-2_ gene may be necessary to identify possible associations with IS*CR*_1_ among the isolates examined in this work. However, the CTX-M-2 subtype is not restricted to animal or even horse isolates. It was previously found in *Salmonella enterica* isolates from the UK and *E. coli* isolates from Brazil originating from clinically ill human patients [39,40].

Ciprofloxacin-resistant animal isolates (55.7%) showed higher prevalence of PMQR-encoding genes compared to human isolates (33.6%). In addition, four *qnr*-variants (*qnrA, qnrB, qnrD* and *qnrS*) were identified in the group of animal isolates, compared to only *qnrB* and *qnrS* among the human isolates. All identified *qnr* variants have already been identified in both groups, however no study is consistent with the results found here [41,42].

Another interesting result is the low prevalence of *bla*_SHV-1_ among the *K. pneumoniae* isolates (10.3% of the human and in 29.4% of the animal *K. pneumoniae* isolates). Even though it is widely considered a plasmid-encoded enzyme, it has been found at high frequencies in *K. pneumoniae*. Further the *bla*_SHV-1_ origin is suspected in the chromosome of *K. pneumoniae*[43]. As some authors indicate high frequencies of up to 90% in *K. pneumoniae* isolates, our data does not seem to fit into the picture [43]. Yet there are other studies showing also low prevalences [44]. Based on these data it could be a local phenomenon.

The β-lactamase genes *bla*_SHV-1_, *bla*_SHV-5_, *bla*_SHV-11_ and *bla*_TEM-52_ were restricted to isolates from inpatients. This fact could be explained by their ancestry, since these genes are frequently found in isolates from hospitals [45].

Combination of various resistance genes was the rule rather than the exception (Table 4). In 70.1% of all isolates two or more genes responsible for β-lactam or quinolone resistance were identified. Combinations of several β-lactamase genes are commonly observed regularly within genomes of Gram-negative bacterial pathogens [46,47] and co-selection of β-lactamase genes and determinants for resistance against other antibiotics e.g. fluoroquinolones has been postulated as a possible mechanism responsible for the widespread distribution of those combined genes [48]. The frequently observed combination of *bla*_CTX-M-15_ and *aac(‘6)-Ib-cr* could provide genetic support for this theory, which has been noted before [49,50]. For this study the presence of *bla*_OXA-48_ in combination with at least two penicillinase genes e.g. TEM, SHV or OXA, a PMQR gene and even sometimes with a CTX-M gene is alarming. Even more worrying is the fact that almost all isolates identified with the OXA-48 carbapenemase originate from companion animals. However, other carbapenemases could be present among the human and animal isolates, which were not tested for. The carbapenem-resistant animal isolates were mainly *K. pneumoniae* and *Enterobacter cloacae* isolates. An unrelated study conducted in our geographic region also detected carbapenem-resistant *Klebsiella* spp. among isolates from dogs [26].

*E. coli* and *K. pneumoniae* are the main species associated with β-lactamase production among isolates resistant to third generation cephalosporins in Germany and other members of the European Union [51]. Data from this study underlines this predominance of both bacterial species as a carrier of ESBL-encoding resistance genes with 74% *E. coli* and 17.5% *K. pneumoniae* isolates in total. Interestingly, the phylogenetic group B2 was strongly underrepresented among the ESBL-*E. coli* isolates from animals. Compared to the human isolates (26.2%) it was only present in 2.4%. The origins of primary sample material could provide an explanation for this discrepancy. Several studies suggest dominance of certain phylogenetic groups in particular specimen and types of infection [52,53]. Zhang et al. demonstrated clustering of phylogenetic group B2 among urinary *E. coli* isolates from human patients [52]. The fact that 53% of the human isolates in this study had been recovered from urine while only 19% were from rectal swabs suggests an association between higher numbers of B2-*E. coli* isolates and the urinary tract of humans. The main source for the animal isolates was faeces (51%) from animals suffering from enteritis, diarrhoea and colic. It is not known whether the ESBL isolates detected were indeed causative for the syndromes observed as they could also be commensal strains that were selectively isolated solely on the basis of their resistance phenotype. This could explain the strong presence of phylogenetic group A/B1 and almost complete lack of group B2. Nevertheless, the pathogenic potential of ESBL-encoding isolates remains uncertain and needs further research. Studies performed on *E. coli* harbouring β-lactamases revealed several virulence-associated traits such as toxins, fimbriae, siderophores, polysaccharide coatings and invasins [54,55]. In addition, patients with bacteremic urinary tract infections (UTIs) caused by ESBL-producing *Enterobacteriaceae* also have prolonged hospitalization and higher antibiotic costs [56].

Our study has limitations with regard to the selection of isolates. Human isolates originate from screening of patients upon admission for carrier-status as well as from clinically ill patients, whereas animal isolates only derive from diseased animals. In addition, selection criteria for ESBL- or PMQR-carrying isolates might have introduced bias. A screening breakpoint of >1 mg/L is recommended for cefotaxime in accordance with the guidelines issued by EUCAST [9]. Usage of the higher concentration of 2 mg/L might have led to loss of some ESBL types. PMQR-genes typically induce low-level quinolone-resistance with ciprofloxacin MIC of ≥ 0.25 μg/mL [57]. For this reason some PMQR may not have been detected due to the MIC of 1 μg/mL used.

Notwithstanding, the study gives an overall view on current dissemination of ESBL-encoding *Enterobacteriaceae* isolates within the region investigated. Further studies should be directed to examining specific transmission pathways assessing the exchange between the human and animal populations in owner-pet studies. In order to address this aspect in more detail, simultaneous screening of pets and/or owners positive for ESBL-producing *Enterobacteriaceae* should be carried out in the future. Another important factor for possible transmission or spread is the environment. Multiresistant bacteria are isolated with increasing frequencies from soil or water and wildlife [58]. Some studies implicate ESBL-producing *E. coli* already as a form of environmental pollution [59]. Our findings are supported by other non-regional studies that confirm the data presented [4,60,61]. In line with this work several studies describe presence of ESBL producing *Enterobacteriaceae* among human and animal samples with dominance of the CTX-M subtype [1-3].

Other aspects such as the colonisation and persistence in humans, animals and the environment as well as host species adaption and interspecies transmissibility of the bacteria involved must be taken into account. As plasmids are epidemiologically relevant agents in ESBL transfer, their role and association with sequence types (STs) need to be examined. Transfer of similar plasmids from human to animal bacterial populations and vice versa has been shown in broilers and broiler farmers in the Netherlands [62] and suggest that a similar situation could also exist between pet and owner.

## Conclusions

The results from this study substantiate the alarming occurrence and ongoing spread of various ESBL-producing, multiresistant *Enterobacteriaceae* strains in the human and animal populations within a defined geographic region in central Hesse, Germany. Our data confirm that companion animals represent novel reservoirs of carbapenemase-producing strains of *K. pneumoniae* and *Enterobacter cloacae*. The study strongly supports the One Health concept which expands interdisciplinary collaborations and communications in all aspects pertaining to the health care for humans and animals [63].

### Ethical approval

The study was approved by the ethics committee of medical faculty of the Justus Liebig University of Giessen (AZ: 95/11).

## Competing interests

The authors declare that they have no competing interests.

## Authors’ contributions

CI, RB, ED and TC designed the study, JS and LF performed the experiments, JS, LF, CI, RB and TC analysed the data, JS, LF, CI, RB, ED and TC wrote the manuscript which was corrected and approved by all the other co-authors.

## References

[B1] Liebana E Carattoli A Coque TM Hasman H Magiorakos AP Mevius D Peixe L Poirel L Schuepbach-Regula G Torneke K Torren-Edo J Torres C Threlfall J Public health risks of enterobacterial isolates producing extended-spectrum-lactamases or AmpC-lactamases in food and food-producing animals: An EU perspective of epidemiology, analytical methods, risk factors, and control options Clin Infect Dis 2013 56 1030 1037 23243183 10.1093/cid/cis1043

[B2] Dolejska M Villa L Hasman H Hansen L Carattoli A Characterization of IncN plasmids carrying blaCTX-M-1 and qnr genes in *Escherichia coli* and *Salmonella* from animals, the environment and humans J Antimicrob Chemother 2013 68 333 339 23060365 10.1093/jac/dks387

[B3] Ewers C Bethe A Semmler T Guenther S Wieler LH Extended-spectrum β-lactamase-producing and AmpC-producing *Escherichia coli* from livestock and companion animals, and their putative impact on public health: a global perspective Clin Microbiol Infect 2012 18 646 655 22519858 10.1111/j.1469-0691.2012.03850.x

[B4] Wu G Day MJ Mafura MT Nunez-Garcia J Fenner JJ Sharma M van Essen-Zandbergen A Rodríguez I Dierikx C Kadlec K Comparative analysis of ESBL-positive *Escherichia coli* isolates from animals and humans from the UK, the Netherlands and Germany PLoS One 2013 8 e75392 24086522 10.1371/journal.pone.0075392 PMC3784421

[B5] Ewers C Grobbel M Stamm I Kopp PA Diehl I Semmler T Fruth A Beutlich J Guerra B Wieler LH Guenther S Emergence of human pandemic O25:H4-ST131 CTX-M-15 extended-spectrum-β-lactamase-producing *Escherichia coli* among companion animals J Antimicrob Chemother 2010 65 651 660 20118165 10.1093/jac/dkq004

[B6] Ewers C Bethe A Stamm I Grobbel M Kopp PA Guerra B Stubbe M Doi Y Zong Z Kola A Schaufler K Semmler T Fruth A Wieler LH Guenther S CTX-M-15-D-ST648 *Escherichia coli* from companion animals and horses: another pandemic clone combining multiresistance and extraintestinal virulence? J Antimicrob Chemother 2014 69 1224 1230 24398338 10.1093/jac/dkt516

[B7] Friese A Schulz J Laube H von Salivati C Hartung J Roesler U Faecal occurrence and emissions of livestock-associated methicillin-resistant *Staphylococcus aureus* (laMRSA) and ESBL/AmpC-producing *E. coli* from animal farms in Germany Berl Munch Tierarztl Wochenschr 2013 126 175 180 23540202

[B8] Meyer E Gastmeier P Kola A Schwab F Pet animals and foreign travel are risk factors for colonisation with extended-spectrum β-lactamase-producing *Escherichia coli* Infection 2012 40 685 687 22971936 10.1007/s15010-012-0324-8

[B9] EUCAST 2012 EUCAST guidelines for detection of resistance mechanisms and specific resistances of clinical and/or epidemiological importance [http://www.eucast.org/fileadmin/src/media/PDFs/EUCAST_files/Consultation/EUCAST_guidelines_detection_of_resistance_mechanisms_121222.pdf]

[B10] Mshana SE Imirzalioglu C Hossain H Hain T Domann E Chakraborty T Conjugative IncFI plasmids carrying CTX-M-15 among *Escherichia coli* ESBL producing isolates at a university hospital in Germany BMC Infect Dis 2009 9 97 19534775 10.1186/1471-2334-9-97 PMC2708165

[B11] Grobner S Linke D Schutz W Fladerer C Madlung J Autenrieth IB Witte W Pfeifer Y Emergence of carbapenem-non-susceptible extended-spectrum –beta-lactamase-producing *Klebsiella pneumoniae* isolates at the university hospital of Tubingen, Germany J Med Microbiol 2009 58 912 922 19502377 10.1099/jmm.0.005850-0

[B12] Kiratisin P Apisarnthanarak A Laesripa C Saifon P Molecular characterization and epidemiology of extended-spectrum- β-lactamase-producing *Escherichia coli* and *Klebsiella pneumoniae* isolates causing health care-associated infection in Thailand, where the CTX-M family is endemic Antimicrob Agents Chemother 2008 52 2818 2824 18505851 10.1128/AAC.00171-08 PMC2493136

[B13] Castillo B Vinué L Román EJ Guerra B Carattoli A Torres C Martínez-Martínez L Molecular characterization of multiresistant *Escherichia coli* producing or not extended-spectrum β-lactamases BMC Microbiol 2013 13 84 23586437 10.1186/1471-2180-13-84 PMC3637601

[B14] Chen X Zhang W Pan W Yin J Pan Z Gao S Jiao X Prevalence of qnr, aac(6’)-Ib-cr, qepA, and oqxAB in *Escherichia coli* Isolates from humans, animals, and the environment Antimicrob Agents Chemother 2012 56 3423 3427 22391545 10.1128/AAC.06191-11 PMC3370760

[B15] Cattoir V Poirel L Nordmann P Plasmid-mediated quinolone resistance pump QepA2 in an *Escherichia coli* isolate from France Antimicrob Agents Chemother 2008 52 3801 3804 18644958 10.1128/AAC.00638-08 PMC2565908

[B16] Cattoir V Poirel L Rotimi V Soussy CJ Nordmann P Multiplex PCR for detection of plasmid-mediated quinolone resistance qnr genes in ESBL-producing enterobacterial isolates J Antimicrob Chemother 2007 60 394 397 17561500 10.1093/jac/dkm204

[B17] Wang M Guo Q Xu X Wang X Ye X Wu S Hooper DC New plasmid-mediated quinolone resistance gene, qnrC, found in a clinical isolate of *Proteus mirabilis* Antimicrob Agents Chemother 2009 53 1892 1897 19258263 10.1128/AAC.01400-08 PMC2681562

[B18] Cavaco LM Hasman H Xia S Aarestrup FM qnrD, a novel gene conferring transferable quinolone Resistance in *Salmonella enterica* serovar Kentucky and Bovis morbificans strains of human origin Antimicrob Agents Chemother 2009 53 603 608 19029321 10.1128/AAC.00997-08 PMC2630628

[B19] Park CH Robicsek A Jacoby GA Sahm D Hooper DC Prevalence in the United States of aac(6’)-Ib-cr encoding a ciprofloxacin-modifying enzyme Antimicrob Agents Chemother 2006 50 3953 3955 16954321 10.1128/AAC.00915-06 PMC1635235

[B20] Jones GL Warren RE Skidmore SJ Davies VA Gibreel T Upton M Prevalence and distribution of plasmid-mediated quinolone resistance genes in clinical isolates of *Escherichia coli* lacking extended-spectrum beta-lactamases J Antimicrob Chemother 2008 62 1245 1251 18827034 10.1093/jac/dkn406

[B21] Clermont O Bonacorsi S Bingen E Rapid and simple determination of the *Escherichia coli* phylogenetic group Appl Environ Microbiol 2000 66 4555 4558 11010916 10.1128/aem.66.10.4555-4558.2000 PMC92342

[B22] GENE-E [http://www.broadinstitute.org/cancer/software/GENE-E/]

[B23] Magiorakos AP Srinivasan A Carey RB Carmeli Y Falagas ME Giske CG Harbarth S Hindler JF Kahlmeter G Olsson-Liljequist B Paterson DL Rice LB Stelling J Struelens MJ Vatopoulos A Weber JT Monnet DL Multidrug-resistant, extensively drug-resistant and pandrug-resistant bacteria: An international expert proposal for interim standard definitions for acquired resistance Clin Microbiol Infect 2012 18 268 281 21793988 10.1111/j.1469-0691.2011.03570.x

[B24] Wieler LH Ewers C Guenther S Walther B Lübke-Becker A Methicillin-resistant staphylococci (MRS) and extended-spectrum beta-lactamases (ESBL)-producing *Enterobacteriaceae* in companion animals: Nosocomial infections as one reason for the rising prevalence of these potential zoonotic pathogens in clinical samples Int J Med Microbiol 2011 301 635 641 22000738 10.1016/j.ijmm.2011.09.009

[B25] Rodriguez I Barownick W Helmuth R Mendoza MC Rodicio MR Schroeter A Guerra B Extended-spectrum β-lactamases and AmpC β-lactamases in ceftiofur-resistant *Salmonella enterica* isolates from food and livestock obtained in Germany during 2003–07 J Antimicrob Chemother 2009 64 301 309 19474065 10.1093/jac/dkp195

[B26] Stolle I Prenger-Berninghoff E Stamm I Scheufen S Hassdenteufel E Guenther S Bethe A Pfeifer Y Ewers C Emergence of OXA-48 carbapenemase-producing *Escherichia coli* and *Klebsiella pneumoniae* in dogs J Antimicrob Chemother 2013 68 2802 2808 23833179 10.1093/jac/dkt259

[B27] Woodford N Wareham DW Guerra B Teale C Carbapenemase-producing *Enterobacteriaceae* and non-*Enterobacteriaceae* from animals and the environment: an emerging public health risk of our own making? J Antimicrob Chemother 2014 69 287 291 24092657 10.1093/jac/dkt392

[B28] Fischer J Rodriguez I Schmoger S Friese A Roesler U Helmuth R Guerra B *Salmonella enterica* subsp. *enterica* producing VIM-1 carbapenemase isolated from livestock farms J Antimicrob Chemother 2013 68 478 480 23034713 10.1093/jac/dks393

[B29] Shi W Li K Ji Y Jiang Q Wang Y Shi M Mi Z Carbapenem and cefoxitin resistance of Klebsiella pneumoniae strains associated with porin OmpK36 loss and DHA-1 β-lactamase production Braz J Microbiol 2013 44 435 442 24294234 10.1590/S1517-83822013000200015 PMC3833140

[B30] Livermore DM CTX-M: changing the face of ESBLs in the UK J Antimicrob Chemother 2005 56 451 454 16006451 10.1093/jac/dki239

[B31] Geser N Stephan R Hächler H Occurrence and characteristics of extended-spectrum β-lactamase (ESBL) producing Enterobacteriaceae in food producing animals, minced meat and raw milk BMC Vet Res 2012 8 21 22397509 10.1186/1746-6148-8-21 PMC3319423

[B32] Ilse O Extended-spectrum β-lactamase genes of *Escherichia coli* in chicken meat and humans, the Netherlands Emerg Infect Dis 2011 17 1216 1222 21762575 10.3201/eid1707.110209 PMC3381403

[B33] Hordijk J Schoormans A Kwakernaak M Duim B Broens E Dierikx C Mevius D Wagenaar JA High prevalence of fecal carriage of extended spectrum β-lactamase/AmpC-producing *Enterobacteriaceae* in cats and dogs Front Microbiol 2013 4 242 23966992 10.3389/fmicb.2013.00242 PMC3745002

[B34] Poirel L Nordmann P Ducroz S Boulouis HJ Arne P Millemann Y Extended-spectrum-β-lactamase CTX-M-15-producing *Klebsiella pneumoniae* of sequence type ST274 in companion animals Antimicrob Agents Chemother 2013 57 2372 2375 23422912 10.1128/AAC.02622-12 PMC3632922

[B35] Johns I Verheyen K Good L Rycroft A Antimicrobial resistance in faecal *Escherichia coli* isolates from horses treated with antimicrobials: A longitudinal study in hospitalised and non-hospitalised horses Vet Microbiol 2012 159 381 389 22565010 10.1016/j.vetmic.2012.04.010

[B36] Dierikx CM van Duijkeren E Schoormans AHW van Essen-Zandbergen A Veldman K Kant A Huijsdens XW van der Zwaluw K Wagenaar JA Mevius DJ Occurrence and characteristics of extended-spectrum-β-lactamase- and AmpC-producing clinical isolates derived from companion animals and horses J Antimicrob Chemother 2012 67 1368 1374 22382469 10.1093/jac/dks049

[B37] Smet A Boyen F Flahou B Doublet B Praud K Martens A Butaye P Cloeckaert A Haesebrouck F Emergence of CTX-M-2-producing *Escherichia coli* in diseased horses: evidence of genetic exchanges of blaCTX-M-2 linked to ISCR1 J Antimicrob Chemother 2012 67 1289 1291 22328640 10.1093/jac/dks016

[B38] Ferreira JC Penha Filho RAC Andrade LN Berchieri A Darini ALC Cutler: Detection of chromosomal blaCTX-M-2 in diverse *Escherichia coli* isolates from healthy broiler chickens Clin Microbiol Infect 2014 doi:10.1111/1469-0691.12531. published ahead of print 10.1111/1469-0691.12531 24438126

[B39] Berman H Barberino MG Moreira ED Riley L Reis JN Distribution of strain type and antimicrobial susceptibility of *Escherichia coli* causing meningitis in a large urban setting in Brazil J Clin Microbiol 2014 doi:10.1128/JCM.03104-13. JCM.03104-13; published ahead of print 10.1128/JCM.03104-13 PMC3993653 24523478

[B40] Burke L Hopkins KL Meunier D de Pinna E Fitzgerald-Hughes D Humphreys H Woodford N Resistance to third-generation cephalosporins in human non-typhoidal *Salmonella enterica* isolates from England and Wales, 2010–12 J Antimicrob Chemother 2014 69 977 981 24288030 10.1093/jac/dkt469

[B41] Yang T Zeng Z Rao L Chen X He D Lv L Wang J Zeng L Feng M Liu JH The association between occurrence of plasmid-mediated quinolone resistance and ciprofloxacin resistance in *Escherichia coli* isolates of different origins Vet Microbiol 2014 170 89 96 24553411 10.1016/j.vetmic.2014.01.019

[B42] Poirel L Cattoir V Nordmann P Plasmid-Mediated Quinolone Resistance; Interactions between Human, Animal, and Environmental Ecologies Front Microbiol 2012 3 24 22347217 10.3389/fmicb.2012.00024 PMC3270319

[B43] Chaves J Ladona MG Segura C Coira A Reig R Ampurdanés C SHV-1 beta-lactamase is mainly a chromosomally encoded species-specific enzyme in *Klebsiella pneumoniae* Antimicrob Agents Chemother 2001 45 2856 2861 11557480 10.1128/AAC.45.10.2856-2861.2001 PMC90742

[B44] Mshana SE Hain T Domann E Lyamuya EF Chakraborty T Imirzalioglu C Predominance of Klebsiella pneumoniae ST14 carrying CTX-M-15 causing neonatal sepsis in Tanzania BMC Infect Dis 2013 13 466 24099282 10.1186/1471-2334-13-466 PMC3851032

[B45] Leistner R Sakellariou C Gürntke S Kola A Steinmetz I Kohler C Pfeifer Y Eller C Gastmeier P Schwab F Mortality and molecular epidemiology associated with extended-spectrum β-lactamase production in Escherichia coli from bloodstream infection Infect Drug Resist 2014 7 57 62 24648746 10.2147/IDR.S56984 PMC3958498

[B46] Shahid M Singh A Sobia F Rashid M Malik A Shukla I Khan HM BlaCTX-M, blaTEM, and blaSHV in *Enterobacteriaceae* from North-Indian tertiary hospital: high occurrence of combination genes Asian Pacific J Tropical Med 2011 4 101 105 10.1016/S1995-7645(11)60046-1 21771430

[B47] Munday CJ Predominance and genetic diversity of community- and hospital-acquired CTX-M extended-spectrum β-lactamases in York, UK J Antimicrob Chemother 2004 54 628 633 15294889 10.1093/jac/dkh397

[B48] Cantón R Coque TM The CTX-M β-lactamase pandemic Curr Opin Microbiol 2006 9 466 475 16942899 10.1016/j.mib.2006.08.011

[B49] Sabtcheva S Kaku M Saga T Ishii Y Kantardjiev T High prevalence of the aac(6’)-Ib-cr gene and its dissemination among *Enterobacteriaceae* Isolates by CTX-M-15 plasmids in Bulgaria Antimicrob Agents Chemother 2009 53 335 336 19001110 10.1128/AAC.00584-08 PMC2612178

[B50] Shibl AM Al-Agamy MH Khubnani H Senok AC Tawfik AF Livermore DM High prevalence of acquired quinolone-resistance genes among *Enterobacteriaceae* from Saudi Arabia with CTX-M-15 β-lactamase Diagn Microbiol Infect Dis 2012 73 350 353 22633335 10.1016/j.diagmicrobio.2012.04.005

[B51] European center for disease prevention and control (2012): Antibiotic resistance surveillance in Europe 2012 [http://www.ecdc.europa.eu]

[B52] Zhang L Foxman B Marrs C Both urinary and rectal *Escherichia coli* isolates are dominated by strains of phylogenetic group B2 J Clin Microbiol 2002 40 3951 3955 12409357 10.1128/JCM.40.11.3951-3955.2002 PMC139655

[B53] Picard B Garcia JS Gouriou S Duriez P Brahimi N Bingen E Elion J Denamur E The Link between phylogeny and virulence in *Escherichia coli* extraintestinal infection Infect Immun 1999 67 546 553 9916057 10.1128/iai.67.2.546-553.1999 PMC96353

[B54] Karisik E Ellington MJ Livermore DM Woodford N Virulence factors in *Escherichia coli* with CTX-M-15 and other extended-spectrum -lactamases in the UK J Antimicrob Chemother 2007 61 54 58 17981835 10.1093/jac/dkm401

[B55] Valat C Auvray F Forest K Metayer V Gay E Garam C Madec JY Haenni M Phylogenetic grouping and virulence potential of extended-spectrum-β-lactamase-producing *Escherichia coli* strains in cattle Appl Environ Microbiol 2012 78 4677 4682 22522692 10.1128/AEM.00351-12 PMC3370483

[B56] Yang YS Ku CH Lin JC Shang ST Chiu CH Yeh KM Lin CC Chang FY Impact of extended-spectrum β-lactamase-producing *Escherichia coli* and *Klebsiella pneumoniae* on the outcome of community-onset bacteremic urinary tract infections J Microbiol Immunol Infect 2010 43 194 199 21291846 10.1016/S1684-1182(10)60031-X

[B57] Martínez-Martínez L Pascual A Jacoby GA Quinolone resistance from a transferable plasmid Lancet 1998 351 797 799 9519952 10.1016/S0140-6736(97)07322-4

[B58] Hartmann A Locatelli A Amoureux L Depret G Jolivet C Gueneau E Neuwirth C Occurrence of CTX-M producing *Escherichia coli* in soils, cattle, and farm environment in France (Burgundy region) Front Microbiol 2012 3 83 22408639 10.3389/fmicb.2012.00083 PMC3297819

[B59] Guenther S Ewers C Wieler LH Extended-spectrum beta-lactamases producing *E. coli* in wildlife, yet another form of environmental pollution? Front Microbio 2011 2 246 10.3389/fmicb.2011.00246 PMC3244693 22203818

[B60] Huber H Zweifel C Wittenbrink MM Stephan R ESBL-producing uropathogenic *Escherichia coli* isolated from dogs and cats in Switzerland Vet Microbiol 2013 162 992 996 23177909 10.1016/j.vetmic.2012.10.029

[B61] Dahmen S Haenni M Chatre P Madec JY Characterization of blaCTX-M IncFII plasmids and clones of *Escherichia coli* from pets in France J Antimicrob Chemother 2013 68 2797 2801 23852541 10.1093/jac/dkt291

[B62] Dierikx C van der Goot J Fabri T van Essen-Zandbergen A Smith H Mevius D Extended-spectrum-β-lactamase- and AmpC-β-lactamase-producing *Escherichia coli* in Dutch broilers and broiler farmers J Antimicrob Chemother 2013 68 60 67 22949623 10.1093/jac/dks349

[B63] Calistri P Iannetti S Danzetta M Narcisi V Cito F Di Sabatino D Bruno R Sauro F Atzeni M Carvelli A Giovannini A The components of ‘one world - one health’ approach Transbound Emerg Dis 2013 60 4 13 24589096 10.1111/tbed.12145

